# Fatty acids in intramuscular fat of Ile de France lambs in two different production systems

**DOI:** 10.5194/aab-61-395-2018

**Published:** 2018-10-26

**Authors:** Milan Margetín, Marta Oravcová, Jana Margetínová, Róbert Kubinec

**Affiliations:** 1 Department for Animal Husbandry Systems, Breeding and Product Quality, National Agricultural and Food Centre – Research Institute of Animal Production Nitra, 95141 Lužianky, Slovakia; 2 Department of Animal Husbandry, Faculty of Agrobiology and Food Resources, Slovak University of Agriculture in Nitra, 94976 Nitra, Slovakia; 3 Institute of Chemistry, Faculty of Natural Sciences, Comenius University in Bratislava, 84215 Bratislava, Slovakia

## Abstract

The fatty acid (FA) composition in the intramuscular fat (IMF) of the musculus longissimus dorsi (MLD) of Ile de France purebred lambs in two different production systems in Slovakia was evaluated using gas chromatography. In the first production system, lambs and ewes were assigned to pasture without access to concentrates (P). In the second system, lambs and ewes were confined indoors with hay/silage and access to concentrates (S). An analysis of variance with the following factors was employed: production system, sex, and production system–sex interactions. The proportions of arachidonic, eicosapentaeonic, docosapentaeonic, and docosahexaenoic FAs, i.e. long-chain polyunsaturated FA (PUFA), were significantly higher in P lambs (1.83, 0.82, 0.92, 0.29 g 100 g${}^{-1}$ FAME, respectively) than in S lambs (0.45, 0.14, 0.30, 0.09 g 100 g${}^{-1}$ FAME, respectively). The proportions of conjugated linoleic acid (CLA), n-6 PUFA, n-3 PUFA, and essential FA (linoleic and α-linolenic) were also significantly higher in P lambs (2.10, 8.50, 4.55, and 8.80 g 100 g${}^{-1}$ FAME, respectively) than in S lambs (0.65, 3.27, 1.50, and 3.64 g 100 g${}^{-1}$ FAME, respectively). The proportions of palmitic acid and myristic acid as important individual saturated FAs (SFA) were significantly higher in S lambs (28.51 and 8.30 g 100 g${}^{-1}$ FAME, respectively) than in P lambs (21.80 and 5.63 g 100 g${}^{-1}$ FAME, respectively). The proportion of all SFAs was also significantly higher in S lambs (57.87 g 100 g${}^{-1}$ FAME) than in P lambs (48.70 g 100 g${}^{-1}$ FAME). From a nutrition and human health point of view (i.e. higher proportions of PUFA, CLA, and essential FAs and lower proportions of SFAs), meat from P lambs was found to be more favourable and would be more highly recommended for consumption.

## 1 Introduction

In Slovakia, the breeding of meat and/or non-dairy dual-purpose sheep breeds that produce heavy lambs (carcass weight above 13 kg) is increasing. The proportion of these breeds ranges from 10 to 15 % of total number of sheep (from 37 to 55 thousand of a total 372 thousand animals in 2017). Lambs are produced using different production systems; however, knowledge about the effects of management and/or feed choice on lamb meat is only known for light lambs in Slovakia, with carcass quality, physicochemical characteristics, and fatty acid (FA) composition of light lambs having been previously studied (Margetín et al., 1993, 2014a, b).

Regardless of lamb type, research around the world is aimed at assessing the quality of lamb meat with respect to evaluating the proportions of essential FAs (linoleic acid and α-linolenic acid) and other human health promoting FAs, such as eicosapentaenoic acid (EPA) and docosahexaenoic acid (DHA). These FAs all belong to the polyunsaturated FAs (PUFA) group and are found in intramuscular and extramuscular fat (Aurousseau et al., 2007a, b; Mortimer et al., 2014; Ponnampalam et al., 2014). The focus of existing research has also been on evaluating the proportion of conjugated linoleic acid (CLA), mainly *cis*-9 *trans*-11 CLA isomer (rumenic acid), known for its anticarcinogenic, anti-atherosclerotic, and antidiabetic effects (Raes et al., 2004; Nuernberg et al., 2005; Schmid et al., 2006; Serra et al., 2009; Gallardo et al., 2014). Regarding saturated FAs (SFA), high proportions of myristic and palmitic acids are assumed to increase the risk of cardiovascular diseases, whilst stearic acid, in comparison, is assumed have no effect on cholesterol levels (Howes et al., 2015). The ratio of PUFA / SFA, in the context of human health, is recommended to be above 0.7, while the ratio of n-6 / n-3 PUFA is recommended to be below 4 (Simopoulos, 2002; Wood et al., 2003; Howes et al., 2015). Furthermore, *cis*-isomers of FAs are assumed to have desirable health effects, whereas *trans*-isomers of FAs are assumed have undesirable health effects (EFSA, 2010), except for those found in products from ruminants (Howes et al., 2015).

Many studies have aimed at finding the potential benefits regarding the consumption of lamb meat (Mortimer et al., 2014; Ponnampalam et al., 2014). The emphasis of these studies has primarily been on assessing the proportions of n-6 PUFA and n-3 PUFA which are assumed to have anti-inflammatory effects and to help protect against autoimmune diseases (Simopoulos, 2002; McAfee et al., 2010; Swanson et al., 2012). According to Australian and European standards, lamb meat is assumed to be an important source of n-3 PUFA when it contains a minimum of 30 to 40 mg of long-chain n-3 PUFA (EPA or DHA) per 100 g of meat (Ponnampalam et al., 2014; Howes et al., 2015). Fisher et al. (2000), Arsenos et al. (2006), Sinanoglou et al. (2013), and Ponnampalam et al. (2014) have indicated that consumer preferences mainly depend on the eating quality and nutrient value of the meat. Fatty acids occurring in fat contribute to the nutrient value of meat; however, the proportions of these FAs depend on the production system and the lamb feed used. Díaz et al. (2002, 2005), Aurousseau et al. (2004, 2007a, b), Nuernberg et al. (2005, 2008), Daley et al. (2010), and Howes et al. (2015) have reported that the FA composition in milk and meat depends on both pasture and concentrates. FA composition may also depend on genotype, although the genetic component of phenotypic variance is believed to be of lesser influence (Santos-Silva et al., 2002; Komprda et al., 2012; Ponnampalam et al., 2014).

In the 1970s and 1980s, Ile de France, as a dual-purpose breed, was mainly kept in order to improve the meat and wool production of traditional breeds in Slovakia. However, since the 1990s the Ile de France breed has been oriented to meat production; lamb feeding systems based on hay/silage and concentrates and systems based on grazing have both been considered. Differences in diet have enabled lamb birth to be postponed from winter to spring and natural pasture to be used. However, until now, no study has aimed at comparing the FA composition between grazing lambs and lambs fed with hay/silage and concentrates in Slovakia. Therefore, the objective of this study was to compare the FA composition in the intramuscular fat separated from the musculus longissimus dorsi (MLD) between Ile de France lambs from the two abovementioned production systems.

## 2 Material and methods

### 2.1 Animals and production systems

A total of 40 Ile de France purebred lambs kept either on pasture (P) or in a stable (S) were included in this experiment. Each group included 20 lambs: 13 males and 7 females. In the first production system, P lambs were born indoors (in April) and were moved to pasture shortly after parturition; the lambs were not separated from the ewes and were allowed to suckle milk. The lambs and ewes in this group were not fed on concentrates or hay/silage. The pasture was natural and was enriched with *Poa pratensis, Festuca rubra, Lolium perenne, Trifolium pratense, Trifolium repens*, and *Lolium multiflorum*, which are dietary sources of conjugated linoleic acid and other polyunsaturated acids according to Mel'uchová et al. (2008, 2009). The stocking rates for this group followed the standards (fence system, eight ewes with their lambs per hectare, as in Sharrow et al., 1981). In the second production system, S lambs were born indoors (in December/January) and housed with the ewes. The ewes' diet consisted of 1 kg maize and 1 kg alfalfa silage, in addition to 2 kg hay and 100–200 g of concentrate (ground barley and maize) per head per day. Approximately 2 weeks after birth, the lambs were moved to permanently opened nurseries, fed with commercial starter (NL min 190.0 g kg${}^{-1}$; fibre max 90.0 g kg${}^{-1}$; fat max 60.0 g kg${}^{-1}$; ash max 100 g kg${}^{-1}$; Ca min 11.2 g kg${}^{-1}$; P min 6.1 g kg${}^{-1}$; Na min 2.0 g kg${}^{-1}$ + supplements), allowed to suckle milk, and offered hay ad libitum. Furthermore, from 2 months after birth, lambs were fed with a 200 g mixture of alfalfa and maize silage (ratio 2:1) per head per day.

Lambs were slaughtered in authorised slaughter houses run by the National Agricultural and Food Centre – Research Institute of Animal Production Nitra and the Slovak University of Agriculture in Nitra (identical slaughtering procedure). The average weights of P and S lambs at slaughter were 29.23 ± 8.20 and 32.2 ± 2.62 kg, respectively, and the average respective ages at slaughter were 106 days and 108 days. The average daily gains were 0.242 ± 0.0341 kg (P lambs) and 0.264 ± 0.0293 kg (S lambs).

The animals used in this study were treated in accordance with the current regulations and standards issued by the Ministry of Agriculture and Rural Development of the Slovak Republic. Manipulation methods were in accordance with the ICLAS Ethics and Animal Welfare Committee.

### 2.2 Analysis of fatty acids

Twenty-four hours after slaughter, meat samples were taken from the MLD between the 9 and 13th vertebra. The analysis of the fatty acid (FA) composition in the intramuscular fat (IMF) of the MLD was undertaken in the laboratory of the Institute of Chemistry (Faculty of Natural Sciences) at Comenius University in Bratislava. After removing the epimysium, 100 g of the MLD was minced, vacuum packed, and stored at -25 ${}^{\circ }$C until lipid analyses were carried out. The proportion of the individual FAs was analysed using capillary gas chromatography (GC). The lipids from 0.5 g meat samples were extracted using 2 mL chloroform–methanol mixtures (2:1 v/v) for 1 h on a rotary shaker. Next, 1 mL of saline water (0.9 % NaCl in water) was added for better separation of the chloroform layer, and the sample was centrifuged at 2000 g for 5 min. The lower 1 mL of the chloroform layers containing the extracted lipids was filtered through anhydrous sodium sulfate and dried under a nitrogen stream at 40 ${}^{\circ }$C. For the preparation of the FA methyl esters (FAME), the base-catalysed methylation procedure with a sodium methoxide in methanol was used. A 1 mL amount of n-hexane was added to the dried chloroform extract in a 2 mL autosampler vial and mixing was resumed. Next, 100 µL of sodium methoxide (0.5 M solution in methanol) was added, mixed, and the vial contents were allowed to react for 15 min at 40 ${}^{\circ }$C with occasional mixing. The vial was cooled at - 20 ${}^{\circ }$C for 10 min, before 60 µL of oxalic acid (0.5 g in 15 mL diethyl ether) was added and mixed thoroughly. The vial was centrifuged to settle the sodium oxalate precipitate, and the FAME solution was used directly for GC analyses. The analyses were performed on a 6890N gas chromatograph (Agilent Technologies, Waldbronn, Germany) with a flame ionisation detector and a 5973 Network mass selective detector. A HP-88 stationary phase capillary column (100 m × 0.25 mm i.d. × 0.2 µm film thickness; J&W Scientific, Agilent Technologies, CA, USA) was used for the FAME separation. The initial column temperature of the programmed run was set to 45 ${}^{\circ }$C and was held for 2 min; this was followed by a step up ramp of 15 ${}^{\circ }$C min${}^{-1}$ to 145 ${}^{\circ }$C and then of 5 ${}^{\circ }$C min${}^{-1}$ to 240 ${}^{\circ }$C and was held for 5 min. Helium was used as the carrier gas with a linear velocity set at 20 cm s${}^{-1}$. Next, 2 µL samples, which represented approximately 10 mg mL${}^{-1}$ FAME, were injected using a 50:1 split at an injection temperature of 300 ${}^{\circ }$C. Separated FAs were identified using reference materials (Supelco 37 Component FAME Mix; PUFA No. 3 from menhaden oil, Sigma-Aldrich, Germany), published retention data, and mass spectrometric measurements. The chromatograms were quantitatively evaluated using an internal normalisation method (the area of each FAME peak in the chromatogram was multiplied by a response factor as a percentage of the total area of all FAME peaks) and published response factors of the flame ionisation detector for FAME (Ackman, 2002). The composition of the FAs was expressed in grams of each individual FAME per 100 g of sum detected FAME. The average relative standard deviation of the FAME analysed with a proportion above 0.5 g 100 g${}^{-1}$ was 1.1 % for the whole analytical procedure and the five replicate samples.

A total of 70 FAs were identified. The hypocholesterolaemic FA/hypercholesterolaemic FA ratio (h/H index) was calculated according to Santos-Silva et al. (2002) and Sinanoglou (2013). The atherogenic index (AI) and thrombogenic index (TI) were calculated according to Ulbricht and Southgate (1991) and Sinanoglou et al. (2013), respectively.

### 2.3 Statistical analysis

Data were evaluated using an analysis of variance and a general linear model procedure as implemented in SAS (2009). Factors including the production system (P lambs and S lambs), sex (males and females), and production system–sex interactions were studied. Least-squares means were compared using a Scheffe test.

**Table 1:** Fatty acids in the intramuscular fat (g 100 g${}^{-1}$ fatty acid methyl esters) of lamb meat dependent on production system and sex.

Fatty acids	Production system		Sex		PxS (P)	SEM	$R^{2}$
	Pasture	Stable	Male	Female			
C12:0 (lauric)	0.75${}^{\text{A}}$	1.06${}^{\text{B}}$	0.94	0.87	0.0174	0.273	0.43
C14:0 (myristic)	5.63${}^{\text{A}}$	8.30${}^{\text{B}}$	6.96	6.97	0.0096	1.307	0.64
C16:0 (palmitic)	21.80${}^{\text{A}}$	28.51${}^{\text{B}}$	25.09	25.22	0.0050	1.496	0.87
C16:1 *cis9* (palmitoleic)	0.47	0.46	0.46	0.49	0.3432	0.063	0.15
C17:0 (margaric)	1.13${}^{\text{A}}$	1.27${}^{\text{B}}$	1.18	1.22	0.1786	0.089	0.51
C18:0 (stearic)	15.65${}^{\text{a}}$	14.51${}^{\text{b}}$	15.29	14.88	0.2847	1.566	0.20
C18:1 *trans9* (elaidic)	0.28	0.28	0.26	0.30	0.3820	0.038	0.19
C18:1 *cis9* (oleic)	24.49${}^{\text{A}}$	28.37${}^{\text{B}}$	25.41${}^{\text{A}}$	27.45${}^{\text{B}}$	0.0459	1.743	0.59
C18:1 *trans11 *(TVA)	4.05${}^{\text{A}}$	1.13${}^{\text{B}}$	2.56	2.62	0.7369	0.439	0.92
C18:2 n-6 (linoleic)	6.42${}^{\text{A}}$	2.73${}^{\text{B}}$	5.00${}^{\text{a}}$	4.14${}^{\text{b}}$	0.0934	1.226	0.75
C18:3 n-6 (GLA)	0.05${}^{\text{A}}$	0.04${}^{\text{B}}$	0.05	0.04	0.0953	0.013	0.37
C18:3 n-3 (ALA)	2.38${}^{\text{A}}$	0.91${}^{\text{B}}$	1.78${}^{\text{A}}$	1.51${}^{\text{B}}$	0.1605	0.274	0.90
C18:2 *cis9 trans11* (RA)	1.82${}^{\text{A}}$	0.56${}^{\text{B}}$	1.06${}^{\text{A}}$	1.33${}^{\text{B}}$	0.0112	0.245	0.88
C20:4 n-6 (arachidonic)	1.83${}^{\text{A}}$	0.45${}^{\text{B}}$	1.43${}^{\text{a}}$	0.85${}^{\text{b}}$	0.1203	0.799	0.54
C20:5 n-3 (EPA)	0.82${}^{\text{A}}$	0.14${}^{\text{B}}$	0.60	0.36	0.1595	0.356	0.57
C22:5 n-3 (DPA)	0.92${}^{\text{A}}$	0.30${}^{\text{B}}$	0.69	0.51	0.2476	0.295	0.60
C22:6 n-3 (DHA)	0.29${}^{\text{A}}$	0.03${}^{\text{B}}$	0.22	0.16	0.4424	0.127	0.45

PxS: interaction between production system and sex; P: probability level; SEM: standard error of mean. TVA: *trans*-vaccenic acid; ALA: α-linolenic acid; GLA: γ-linolenic acid; RA: rumenic acid; EPA: eicosapentaeonic acid; DPA: docosapentaeonic acid; DHA: docosahexaeonic acid. ${}^{\text{A, B}}$: differences at P<0.001; ${}^{\text{a, b}}$: differences at P<0.05.

## 3 Results and discussion

### 3.1 Analysis of fatty acids

#### 3.1.1 Effect of production system

The composition of FAs identified in the IMF of the MLD as affected by production system is shown in Table 1. This factor significantly influenced the proportions of almost all of the FAs that were investigated. The prevailing individual saturated FAs (SFA) were palmitic acid (PA), stearic acid (SA), and myristic acid (MA). A crucial finding in this study was the significantly lower (P<0.001) proportion of PA (which has undesirable effects on human health) in P lambs (21.80 g 100 g${}^{-1}$ FAME) in comparison with S lambs (28.51 g 100 g${}^{-1}$ FAME) as well as the significantly lower (P<0.001) proportion of MA (which also has undesirable effects on human health) in P lambs (5.63 g 100 g${}^{-1}$ FAME) compared with S lambs (8.30 g 100 g${}^{-1}$ FAME). The proportion of SA (which has a neutral effect on total cholesterol) was significantly higher (P<0.05) in P lambs compared with S lambs (15.65 vs. 14.51 g 100 g${}^{-1}$ FAME); however, the difference in SA between P and S lambs was relatively lower than the difference in PA and MA between lambs from the two respective production systems. When compared with values of PA, MA, and SA from the literature, a lower proportion of PA for Ile de France grazing lambs (20.7 g 100 g${}^{-1}$FAME) than for lambs fed with concentrate supplement (23.9 g 100 g${}^{-1}$FAME) was also reported by Aurousseau et al. (2007a). The high proportion of MA in S lambs (8.30 g 100 g${}^{-1}$ FAME) can be attributed to a diet consisting of a relatively high portion of maize. When feed contains more energy, the intake of energy is higher and consequently de novo synthesis of MA and PA is also higher (Aurousseau et al., 2007a). Regarding MA in S lambs, Díaz et al. (2005) reported both higher and lower proportions of MA (from 5.73 to 10.45 g 100 g${}^{-1}$ FAME) for heavy lambs of various breeds and crosses fed on either grass, combined grass–concentrate, or concentrate in Spain, Germany, Great Britain, and Uruguay (combined effect of breed, diet, sex, and husbandry). Daley et al. (2010) reported that grass-finished beef tends to have higher proportions of cholesterol neutral SA and lower proportions of cholesterol-elevating SFAs (MA and PA). In contrast to findings regarding SA in this study (15.65 vs. 14.51 g 100 g${}^{-1}$ FAME in P and S lambs), Cividini et al. (2014) observed no statistical difference in the proportion of SA between pastured (14.69 vs. 16.93 g 100 g${}^{-1}$ FAME) and stabled lambs (15.00 vs. 15.08 g 100 g${}^{-1}$ FAME). Fiori et al. (2013) reported higher proportions of PA (23.47 vs. 25.07 g 100 g${}^{-1}$ FAME) and SA (17.83 vs. 16.76 g 100 g${}^{-1}$ FAME) for Sarda x Ile de France crossbreeds that were either grazing or fed with hay and concentrates, which is in contrast to the P and S lambs from this study. According to Howes et al. (2015), animals on feedlot or grain-based diets produce higher levels of SA and PA than those on pasture-based diets with minimal amounts of grain. In this study, the proportion of margaric acid was 1.13 g 100 g${}^{-1}$ FAME (in the meat of P lambs) and 1.27 g 100 g${}^{-1}$ FAME (in the meat of S lambs), (P<0.001). When comparing these values with the literature, they are more than twofold higher than Costa et al. (2015) reported for Brazilian lambs (0.45 vs. 0.60 g 100 g${}^{-1}$ FAME, P<0.05) fed on a diet comprising 20:80 and 50:50 ratios of forage/concentrate, and slaughtered at live weight of 36 kg.

Regarding individual monounsaturated FAs (MUFA), a lower (P<0.001) proportion of oleic acid (OA) was found in P lambs (24.49 g 100 g${}^{-1}$ FAME) compared with S lambs (28.37 g 100 g${}^{-1}$ FAME). These findings concur with Aurousseau et al. (2007a), who reported that the proportion of OA was 23.4 g 100 g${}^{-1}$FAME for grazing lambs and 31.4 g 100 g${}^{-1}$ FAME for lambs fed on diets including concentrates. Aurousseau et al. (2007a) also indicated that the proportion of OA is mainly affected by feeding. Oleic acid is a FA that is mobilised from body fat, and its higher proportion in S lambs is probably linked to the higher daily gains of these lambs. Furthermore, concentrates in the diets of lambs are expected to increase the absorption of OA, which is found in cereal grains (Jenkins, 1994). Silva Sobrinho et al. (2014) reported higher proportions of OA (40.25 and 39.30 g 100 g${}^{-1}$ FAME) for Ile de France purebred lambs on a diet with a forage/concentrate ratio of 50:50, which was also enriched by various portions of mulberry hay, compared with P and S lambs in this study. No differences (P>0.05) in the proportions of elaidic acid (EA) and palmitoleic acid were found; these proportions were either equal (0.28 g 100 g-1 FAME) or almost equal (0.47 and 0.46 g 100 g-1 FAME). Conversely, Cividini et al. (2014) reported significant differences (P<0.05) in the proportions of EA between pastured and stabled Jezersko–Solčava lambs (0.28 and 0.30 g 100 g${}^{-1}$ FAME vs. 0.17 and 0.22 g 100 g${}^{-1}$ FAME, respectively). The proportion of *trans*-vaccenic acid (TVA), which is the most important precursor of conjugated linoleic acid (CLA), was several-fold higher (P<0.001) in P lambs (4.05 g 100 g${}^{-1}$ FAME) than in S lambs (1.13 g 100 g${}^{-1}$ FAME). These findings agree with Nuernberg et al. (2005), Aurousseau et al. (2007a), and Cividini et al. (2014), who reported a significantly higher proportion of TVA in grass feed lambs than in lambs fed on a diet including concentrates. According to Daley et al. (2010), grass-based diets also enhance total CLA isomers and TVA in beef. Vaccenic acid (VA) in ruminant meat is considered to have an anti-carcinogenic effect (Pariza et al., 2001) and a possible positive role in the endogenous synthesis of CLA (Cividini et al., 2013). The higher proportion of TVA in grazing lambs is probably a result of the fact that VA, among others, originates as a main intermediate during the bio-hydrogenation of α-linolenic acid (ALA), which occurs in grass.

Regarding individual polyunsaturated FAs (PUFA), the proportion of essential linoleic acid (LA) was about twofold higher (P<0.001) in P lambs (6.42 g 100 g${}^{-1}$ FAME) than in S lambs (2.73 g 100 g${}^{-1}$ FAME). The proportion of ALA, which is also an essential FA that is mainly found in pasture feed (Mel'uchová et al., 2009), was about twofold higher (P<0.001) in P lambs (2.38 g 100 g${}^{-1}$ FAME) than in S lambs (0.91 g 100 g${}^{-1}$ FAME). Findings regarding LA and ALA from this study agree with Cividini et al. (2014) but disagree with Aurousseau et al. (2007a) – Aurousseau et al. (2007a) reported the highest proportion of LA in lambs not allowed to graze (6.4 g 100 g${}^{-1}$ FAME). Findings with regards to the higher proportion of ALA in grazing lambs from this study agree with Wood et al. (2008) and Serra et al. (2009); both of these studies indicated that grass is rich in this particular FA. The proportion of rumenic acid (RA) was significantly higher (P<0.001) in P lambs (1.82 g 100 g${}^{-1}$ FAME) than in S lambs (0.56 g 100 g${}^{-1}$ FAME) in this research which agrees with Aurosseau et al. (2007a), although these authors reported a slightly lower difference in RA between grazing and stall lambs (1.1 and 0.7 g 100 g${}^{-1}$ FAME). The proportions of arachidonic acid (AA), eicosapentaenoic acid (EPA), docosapentaeonic acid (DPA), and docosahexaenoic acid (DHA), which are important from a human health point of view, are of great interest; these acids displayed significantly higher proportions (P<0.001) in P lambs (1.83, 0.82, 0.92, 0.29 g 100 g${}^{-1}$ FAME, respectively) than in S lambs (0.45, 0.14, 0.30, 0.09 g 100 g${}^{-1}$ FAME, respectively). These findings agree with Daley et al. (2010) and Howes et al. (2015) who reported that the dietary source of lambs may influence the proportion of these FA in their meat – with grazing lambs showing higher proportions of the abovementioned beneficial FAs. The proportions of AA, EPA, and DPA in P lambs were found to be higher than those reported by Yousefi et al. (2012) for the tailed Zel breed in Iran (0.98, 0.34 and 0.26 g 100 g${}^{-1}$ FAME, respectively). Aurousseau et al. (2007a) also reported higher proportions of AA, EPA, DPA, and DHA for lambs on grass than for lambs on either pasture feeding combined with stall-finishing on concentrates and hay or exclusively on concentrates and hay. The proportions of γ-linolenic acid were 0.05 and 0.04 g 100 g${}^{-1}$ FAME in meat of P and S lambs, respectively, and were about twofold lower when compared with results reported by Cividini et al. (2014) for Jezersko–Solčava lambs (between 0.09 and 0.13 g 100 g${}^{-1}$ FAME) slaughtered at a live weight of between 30 and 38 kg.

#### 3.1.2 Effect of sex

The composition of FAs as affected by sex is shown in Table 1. No significant differences were found in the proportions of MA, PA, SA, lauric, and margaric acids between males and females. Conversely, Arsenos et al. (2006) reported a significant difference in the proportion of SA dependent on sex. Regarding individual MUFA, the highest significant difference (P<0.001) in the proportions of OA was found between males (25.41 g 100 g${}^{-1}$ FAME) and females (27.45 g 100 g${}^{-1}$ FAME). Cividini et al. (2008) reported slightly higher proportions of OA for Jezersko–Solčava female and male lambs (31.90 and 28.37 g 100 g${}^{-1}$ FAME, respectively) and assumed that differences in the composition of this FA between the sexes were due to the different content of intramuscular fat. Both LA and ALA displayed higher proportions in males than females (5.00 vs. 4.14 g 100 g${}^{-1}$ FAME and 1.78 vs. 1.51 g 100 g${}^{-1}$ FAME, respectively), which is probably the result of differences in male and female vitality, voracity, and metabolism (Margetín et al., 2014b). Cividini et al. (2008) also reported higher proportions of LA and ALA for males than for females (8.71 vs. 6.80 g 100 g${}^{-1}$ FAME and 3.31 vs. 2.83 g 100 g${}^{-1}$ FAME, respectively). In addition, significantly different proportions of RA (1.06 vs. 1.33 g 100 g${}^{-1}$ FAME, respectively; P<0.001) and AA (1.43 vs. 0.85 g 100 g${}^{-1}$ FAME, respectively; P<0.05) were found between males and females in this study. The findings of this study regarding the proportions of AA roughly agreed with Cividini et al. (2008): 3.50 g 100 g${}^{-1}$ FAME (males) vs. 2.43 g 100 g${}^{-1}$ FAME (females).

The production system–sex interaction differed significantly (P<0.05; P<0.001) mainly with respect to the proportions of individual SFAs (lauric acid, MA, PA). To the authors' best knowledge, no studies investigating the effect of production system and sex interaction currently exist in the literature; therefore, respective comparisons could not be carried out.

**Table 2:** Fatty acid groups (g 100g${}^{-1}$ fatty acid methyl esters), their ratios, and indexes dependent on production system and sex.

Fatty acids	Production system		Sex		PxS (P)	SEM	$R^{2}$
	Pasture	Stable	Male	Female			
SFA${}^{1}$	48.70${}^{\text{A}}$	57.87${}^{\text{B}}$	53.46	53.11	0.0133	3.041	0.78
MUFA${}^{2}$	34.08	35.53	33.52${}^{\text{A}}$	35.86${}^{\text{B}}$	0.1300	2.306	0.25
PUFA${}^{3}$	17.21${}^{\text{A}}$	6.80${}^{\text{B}}$	5.07	5.23	0.1522	2.971	0.79
*Trans*-UFA${}^{4}$	7.04${}^{\text{A}}$	3.27${}^{\text{B}}$	13.01	11.00	0.9466	0.635	0.91
*Cis*-UFA${}^{5}$	29.28${}^{\text{A}}$	32.36${}^{\text{B}}$	29.59${}^{\text{A}}$	32.05${}^{\text{B}}$	0.1492	2.010	0.47
BCFA (*iso*, *anteiso*)${}^{6}$	1.92${}^{\text{A}}$	2.43${}^{\text{B}}$	2.11	2.24	0.0768	0.194	0.71
Essential FA (LA + ALA)	8.80${}^{\text{A}}$	3.64${}^{\text{B}}$	6.78${}^{\text{a}}$	5.66${}^{\text{b}}$	0.0919	1.447	0.80
n-6 PUFA${}^{7}$	8.50${}^{\text{A}}$	3.27${}^{\text{B}}$	6.64${}^{\text{a}}$	5.14${}^{\text{b}}$	0.0933	2.055	0.69
n-3 PUFA${}^{8}$	4.55${}^{\text{A}}$	1.50${}^{\text{B}}$	3.41${}^{\text{a}}$	2.65${}^{\text{b}}$	0.1667	1.008	0.75
CLA${}^{9}$	2.10${}^{\text{A}}$	0.65${}^{\text{B}}$	1.23${}^{\text{A}}$	1.52${}^{\text{B}}$	0.0121	0.266	0.90
PUFA / SFA	0.36${}^{\text{A}}$	0.12${}^{\text{B}}$	0.26	0.21	0.1062	0.080	0.75
∑n-6 / ∑n-3 PUFA	1.86${}^{\text{A}}$	2.23${}^{\text{B}}$	1.99	2.07	0.1635	0.331	0.22
LA / ALA	2.68	3.06	2.82	2.91	0.1938	0.588	0.11
LC n-6 / LC n-3 PUFA${}^{10}$	0.92	0.92	0.97	0.88	0.3079	0.166	0.10
AI (atherogenic index)	0.97${}^{\text{A}}$	1.58${}^{\text{B}}$	1.30	1.25	0.0031	0.230	0.74
TI (thrombogenic index)	1.24${}^{\text{A}}$	2.13${}^{\text{B}}$	1.68	1.68	0.0224	0.228	0.83
$h/H^{11}$ index	1.38${}^{\text{A}}$	0.90${}^{\text{B}}$	1.17	1.11	0.0076	0.200	0.70

${}^{1}$ SFA is the sum of saturated fatty acids: C8:0 + C10:0 + C11:0 + C12:0 + C13:0 + *iso*-C14:0 + C14:0 + *iso*-C15:0 + *anteiso*-C15:0 + C15:0 + *iso*-C16:0 + C16:0 + *iso*-C17:0 + *anteiso*-C17:0 + C17:0 + *iso*-C18:0 + C18:0 + C19:0 + C20:0 + C21:0 + C22:0;${}^{2}$ MUFA is the sum of monounsaturated FA: C12:1 + C14:1 + tC16:1 + cC16:1 + 9cC16:1 + C17:1 + 6–8tC18:1 + 9tC18:1 + 10tC18:1 + 11tC18:1 + 12tC18:1 + 9cC18:1 + (15t + 11cC18:1) + 12cC18:1 + 13cC18:1 + (14cC18:1 + 9t12t18:2/2) + 15cC18:1 + (C18:2 + C19:1/2) + C20:1;${}^{3}$ PUFA is the sum of polyunsaturated FA: (14cC18:1 + 9t12t18:2/2) + 9c13tC18:2 + (8t13c + 9c12tC18:2) + (9t12c + 11t15cC18:2) + C18:2n-6 + 9c15cC18:2 + 12c15cC18:2 + *cc*C18:2 + *cc*C18:2 + (C18:3 n-6 GLA) + (C18:2+C19:1/2) + cyklo + 9t12c15cC18:3 + C18:3 n-3 + (9c11tC18:2 CLA) + *ct*CLA + *cc*CLA + *tc*CLA + *tt*CLA + C18:3 + C20:2 + C20:3 *n-9* + C20:3 n-6 + C20:4 n-6 + C20:3 n-3 + C20:4 n-3 + C20:5 n-3 + furyl C22 + C22:4 n-3 + C22:5 n-3 + C22:6 n-3;${}^{4}$ *Trans*-UFA is the sum of *trans* UFA: tC16:1 + 6–8tC18:1 + 9tC18:1 + 10tC18:1 + 11tC18:1 + 12tC18:1 + ((15t+11cC18:1/3)*2) + (14cC18:1 + 9t12t18:2/2) + 9c13tC18:2 + (8t13c+9c12tC18:2) + 9t12c+11t15cC18:2 + *tt*CLA;${}^{5}$ *Cis*-UFA is the sum of *cis*-UFA: cC16:1 + 9cC16:1 + 9cC18:1 + (15t+11cC18:1/3) + 12cC18:1 + 13cC18:1 + (14cC18:1 + 9t12tC18:2/2) + 15cC18:1 + 9c15cC18:2 + 12c15c18:2 + *cc*C18:2 + *cc*C18:2 + 9c11tC18:2 CLA + *ct*CLA + *cc*CLA + *tc*CLA;${}^{6}$ BCFA is the sum of *iso and anteiso*-FA: *iso*-C14:0 + *iso*-C15:0 + *anteiso*-C15:0 + *iso*-C16:0 + *iso*-C17:0 + *anteiso*-C17:0 + *iso*-C18:0;${}^{7}$ n-6 PUFA is the sum of n-6 PUFA: C18:2 n-6 + C18:3 n-6 GLA + C20:3 n-6 + C20:4 n-6;${}^{8}$ n-3 PUFA is the sum of n-3 PUFA: C18:3 n-3 + C20:3 n-3 + C20:4 n-3 + C20:5 n-3 + C22:4 n-3 + C22:5 n-3 + C22:6 n-3;${}^{9}$ CLA = 9c11tC18:2 CLA + *ct*CLA + *cc*CLA + *tc*CLA + *tt*CLA;${}^{10}$ LC n-6 PUFA = n-6 PUFA – LA and LC n-3 PUFA = n-3 PUFA – ALA;${}^{11}$ h/H = hypocholesterolaemic FA/hypercholesterolaemic FA. For remaining explanations see Table 1.

### 3.2 Analysis of groups of fatty acids, their ratios, and indexes

#### 3.2.1 Effect of production system

The proportions of FA groups believed to affect human health (Wood et al., 2003; Sinanoglou et al., 2013), their ratios, and indexes are shown in Table 2. Lamb meat is assumed to be highly nutritious and easy digestible (Milewski, 2006; Nuernberg et al., 2008). Some doubts about the health effects of the consumption of red meat (including lamb meat) and its lipid composition have arisen due to the relatively high proportion of SFAs and relatively low proportion of PUFAs (McAfee et al., 2010; Howes et al., 2015). The proportion of the SFA group was significantly higher (P<0.001) in S lambs (57.87 g 100 g${}^{-1}$ FAME) than in P lambs (48.70 g 100 g${}^{-1}$ FAME) in this research. This finding agrees with studies such as Díaz et al. (2005), Aurousseau et al. (2007a), and Yousefi et al. (2012), which found that the dietary source of lambs is the reason for this difference – less SFA was found in grazing lambs. When compared with light lambs, the content of SFA was found to be higher in heavy lambs (in both P and S lambs), most likely due to the fact that light lambs are less fatty. In addition, the lower content of SFA in light lambs probably results from their diet which mainly consists of milk (Lanza et al., 2006; Margetín et al., 2014a).

The proportion of the PUFA group was significantly higher (P<0.001) in P lambs (17.21 g 100 g${}^{-1}$ FAME) than in S lambs (6.80 g 100 g${}^{-1}$ FAME). Díaz et al. (2002, 2005), Aurousseau, et al. (2004, 2007a, b), and Nuernberg et al. (2008) also reported that the proportion of these FAs was higher in lambs on pasture than in lambs fed with hay and concentrates. Moreover, Fiori et al. (2013) found the proportion of the PUFA group to be higher in lambs on pasture (37.92 g 100 g${}^{-1}$ FAME) than in lambs on a diet including concentrates (32.34 g 100 g${}^{-1}$ FAME). The proportion of CLA was significantly higher (P<0.001) in P lambs (2.10 g 100 g${}^{-1}$ FAME) than in S lambs (0.65 g 100 g${}^{-1}$ FAME). This agreed with the fact that grass-based diets were shown to enhance total CLA in meat (Daley et al., 2010). When comparing the findings from this study with Raes et al. (2004), who recommended that CLA in lamb meat be between 0.2 and 1.0 g 100 g${}^{-1}$ FAME, the proportion of CLA in P lambs exceeded (was more than double) the recommended value. The proportion of essential FAs as a sum of LA and ALA was about twofold higher (P<0.001) in P lambs (8.80 g 100 g${}^{-1}$ FAME) than in S lambs (3.64 g 100 g${}^{-1}$ FAME). While branched-chain (BC) FAs mainly occur in milk lipids, it is important to emphasise that meat lipids also consist of these FA (Sinanoglou et al., 2013). The proportion of the BCFA group was found to be significantly lower (P<0.001) in P lambs (1.92 g 100 g${}^{-1}$ FAME) than in S lambs (2.43 g 100 g${}^{-1}$ FAME). According to Serra et al. (2009), BCFAs are indicators of rumen activity, and their proportion in meat increases with lamb weight and age. Findings from this study regarding BCFAs were comparable with those reported by Aurousseau et al. (2007a) for lambs in similar production systems (between 1.3 and 2.2 g 100 g${}^{-1}$ FAME).

The ratios and indexes of FA groups as affected by production system are shown in Table 2. According to Sinanoglou et al. (2013), these ratios and indexes are important when assessing the nutrition value of lipids. The ratio of PUFA / SFA was significantly lower (P<0.001) in S lambs (0.12) than in P lambs (0.36), reflecting fact that PUFAs were significantly lower and SFAs were significantly higher in S lambs. Both ratios were lower than those recommended in the literature (above 0.7 – Raes et al., 2004 and Lanza et al., 2006 and 0.45 – Williams, 2000). Similar to findings from this study, Sanudo et al. (2000) reported PUFA / SFA ratios between 0.14 and 0.33 for Spanish Merino, Rasa Aragonesa, and Welsh Mountain lambs; Yousefi et al. (2012) reported values of 0.19 and 0.16 for lambs of two Iranian breeds. Conversely, Díaz et al. (2005) and Sinanoglou et al. (2013) reported the ratios of PUFA / SFA to be slightly higher (0.38, 0.20, 0.19, and 0.21 or between 0.37 and 0.49) than those found in P and S lambs. The n-6 / n-3 PUFA ratios were 1.86 (P lambs) and 2.23 (S lambs). Williams (2000) indicated that this ratio plays a significant role regarding the risk of atherosclerosis. A low n-6 / n-3 PUFA ratio reduces the risk of many chronic diseases that mainly occur in Europe and North America populations (Simopoulos et al., 2008). The recommended n-6 / n-3 PUFA ratio value is a maximum of 4 (Raes et al., 2004; Howes et al., 2015); therefore, the values found in this study are desirable. When assessing the cholesterolaemic effect of lipids, the h/H index may be useful due to the fact that it is related to cholesterol metabolism (Santos-Silva et al., 2013; Raes et al., 2003; Sinanoglou et al., 2013). The values of the h/H index significantly differed (P<0.001) between P lambs (1.30) and S lambs (0.90) in this study. In contrast, Santos-Silva et al. (2002) found no statistical difference (P>0.05) in the h/H index which ranged from 1.84 to 2.04; however, the value of this index also tended to be higher in lambs on pasture. The values of AI and TI significantly differed (P<0.001) between P lambs (0.97 and 1.24) and S lambs (1.58 and 2.13). According to Sinanoglou et al. (2013), AI and TI should not exceed 1.0. The values of AI found for P lambs agree with Vacca et al. (2008), who reported an AI value close to 1.0 for Sarda purebred and crossbred lambs; however the findings from this study were lower than Oriani et al. (2005), who reported a value of 1.35 for Merino lambs, and higher than Sinanoglou et al. (2013), who reported values from 0.55 to 0.73 for lambs from Greek breeds. Therefore, with respect to AI, the meat of P lambs would be considered a healthy food with a desirable FA composition, and its composition may help prevent cardiovascular diseases in consumers. The values of TI found in both P and S lambs were either higher than those reported by Vacca et al. (2008) and Sinanoglou et al. (2013), who mentioned TI values between 0.9 and 1.1 and between 0.83 and 1.21, respectively, or were lower than those reported by Oriani et al. (2005), who mentioned a TI value of 1.69. However, Vacca et al. (2008), Sinanoglou et al. (2013), and Oriani et al. (2005) all evaluated the meat of suckling lambs.

#### 3.2.2 Effect of sex

The proportions of the FA groups, their ratios, and indexes in dependence on sex are shown in Table 2. The significant differences between males and females (P<0.001; P<0.05) were seen in the proportions of CLA, MUFA, *cis*UFA, n-3 PUFA, and n-6 PUFA. Similarly to individual FAs, the differences in the proportions of FA groups may be explained by different male and female feeding behaviour and metabolism that results in differences in fat content. Arsenos et al. (2006) reported the significant effect (P<0.05) of sex in only one (PUFA) out of four FA groups (SFA, UFA, MUFA, PUFA). Cividini et al. (2008) reported significant differences between males and females in MUFA, PUFA, n-6 PUFA, and n-3 PUFA. Similarly to this study (MUFA higher in females), the proportion of MUFA was found to be higher in Jezersko–Solčava females than in males. However, in contrast to this study (no significant differences between sexes), the proportion of PUFA was found to be higher in Jezersko–Solčava males than in females.

In a similar fashion to the analyses of individual SFAs, the production system–sex interaction had a significant influence (P<0.05) on the proportion of SFAs (considered as a group). Production system–sex interaction also had a significant influence on AI, TI, and the h/H index. However, as no studies investigating the effect of production system–sex interaction currently exist in the literature, respective comparisons could not be carried out.

## 4 Conclusions

From a nutrition and human health standpoint (with respect to higher proportions of PUFAs, e.g. mainly LC n-3 PUFA, CLA, and essential FA, and lower proportions of SFA), the meat of lambs assigned to pasture feeding with no concentrate supplement seems to be better than the meat of lambs assigned to grow in a stable with a diet based on hay, silage, and concentrates. Therefore, consumers are recommended to favour the meat of grazing lambs in spite of the fact that the market price of this meat may be higher owing to its higher quality.

## Data Availability

Data are available from the corresponding author upon request.
